# Histamine and gamma-aminobutyric acid in the nervous system of *Pygospio elegans* (Annelida: Spionidae): structure and recovery during reparative regeneration

**DOI:** 10.1186/s40850-022-00160-7

**Published:** 2022-12-07

**Authors:** Zinaida I. Starunova, Ksenia V. Shunkina, Elena L. Novikova, Viktor V. Starunov

**Affiliations:** 1 Zoological Institute RAS, 199034, Universitetskaya nab. 1, St. Petersburg, Russia; 2 St. Petersburg State University, 199034, Universitetskaya nab. 7-9, St. Petersburg, Russia

**Keywords:** Histamine, Gamma-aminobutyric acid, Annelida, *Pygospio elegans*, Confocal microscopy, Nervous system, Regeneration

## Abstract

**Background:**

In recent two decades, studies of the annelid nervous systems were revolutionized by modern cell labeling techniques and state-of-the-art microscopy techniques. However, there are still huge gaps in our knowledge on the organization and functioning of their nervous system. Most of the recent studies have focused on the distribution of serotonin and FMRFamide, while the data about many other basic neurotransmitters such as histamine (HA) and gamma-aminobutyric acid (GABA) are scarce.

**Results:**

Using immunohistochemistry and confocal microscopy we studied the distribution of histamine and gamma-aminobutyric acid in the nervous system of a spionid annelid *Pygospio elegans* and traced their redevelopment during reparative regeneration. Both neurotransmitters show specific patterns in central and peripheral nervous systems. HA-positive cells are concentrated mostly in the brain, while GABA-positive cell somata contribute equally to brain and segmental ganglia. Some immunoreactive elements were found in peripheral nerves. Both substances were revealed in high numbers in bipolar sensory cells in the palps. The first signs of regenerating HAergic and GABAergic systems were detected only by 3 days after the amputation. Further redevelopment of GABAergic system proceeds faster than that of HAergic one.

**Conclusions:**

Comparisons with other annelids and mollusks examined in this respect revealed a number of general similarities in distribution patterns of HAergic and GABAergic cells in different species. Overall, the differences in the full redevelopment of various neurotransmitters correlate with neuronal development during embryogenesis. Our results highlight the importance of investigating the distribution of different neurotransmitters in comparative morphological and developmental studies.

## Background

The studies of annelid neuroanatomy have reached new heights and broadened in recent two decades. The neuromorphology of different annelid groups has been revealed in great detail, which, together with new phylogenomic data, have resulted in a reconsideration of some former theories of the nervous system evolution in the phylum [1–3]. However, our knowledge about some aspects of annelid neuroanatomy is still incomplete. The first gap is an insufficient taxon sampling with modern microscopic techniques, which hinders a comprehensive comparative analysis. The second gap is the incompleteness of the data on the groups already examined in some respects. After the advent of confocal microscopy into zoological research, only a restricted number of antibodies against neuroactive substances and structural proteins has been used. The majority of modern studies of the annelid nervous system have employed antibodies against acetylated α-tubulin, serotonin, and FMRFamide [4–13]. Acetylated α-tubulin is a cytoskeletal protein characteristic of neuronal microtubules and ciliary structures [14, 15]. It is widely used to highlight the overall scaffold of the nervous system [16]. Serotonin is a monoamine that acts as an important signal molecule in various processes such as the regulation of muscle contraction, sensory processes and regeneration of the central nervous system (CNS) and neurogenesis [17–19]. FMRFamide is a neuropeptide first described as a cardio excitatory peptide from the bivalve mollusk *Macrocallista nimbosa* [20]. Later on, a number of FMRFamide-like peptides were described [21, 22]. There is evidence that anti-FMRFamide antibodies can bind with structurally related RFamides [23].

Little is known about the distribution of the basic neuroactive substances other than serotonin and FMRFamide in annelids. Most studies focused on different neuropeptides. The antibodies developed against conserved neuropeptide motifs DLamide, FVamide, FLamide, GWamide, and RYamide have shown cross-species reactivity in various marine invertebrates [24]. The immunostained cells had projections to larval ciliary bands, indicating a conserved role of neuropeptides in the regulation of ciliary locomotion. In *Platynereis* a full neuropeptide complement was characterized, and for some peptides specific antibodies were raised to visualize their distribution in development [25, 26]. The same antibodies were successfully used in a study of the brain anatomy of the Dinophilidae [27]. Surprisingly, a high variation in the expression patterns of neuropeptides across species was revealed, suggesting a high plasticity in neuropeptide expression.

Other neurotransmitters, in particular, histamine (HA) and γ-aminobutyric acid (GABA), have been studied rather poorly in annelids. This is unfortunate, because these neuroactive substances, widely distributed in the animal kingdom, play a crucial role both in physiology and in behavior. Histamine is a biogenic monoamine, which is known to be a neurotransmitter as well as an inflammatory modulator [28]. HA-containing cells are common in the CNS of different invertebrates, with HA-immunoreactive elements playing an important role in the visual, mechanosensory, and vestibular sensory systems in mollusks [29–31], annelids [32], and arthropods [33]. Histamine, a humoral factor in inflammation, has also been found in extracts of earthworms *Lumbricus terrestris* and *Eisenia fetida* [34]. Cellular inflammatory responses in annelids are comparable to the inflammatory process in vertebrates [34].

Gamma-aminobutyric acid (GABA) is a neurotransmitter, widely represented in the nervous system of mammals and various groups of invertebrates [35]. In *Hydra vulgaris*, GABA decreases the activity of impulse-generating systems [36], while in the flatworm *Gastrothylax crumenifer* it has a general inhibitory effect [37]. The presence of GABA in the nervous tissues of *Nereis* [38] and *Lumbricus* [39] was demonstrated for the first time by autoradiographic techniques and paper chromatographic methods. 5-hydroxytryptamine (5-HT) and GABA regulate the muscle activity of the body wall in annelids and nematodes [40]. Research on cerebral brain extirpation [41] and embryogenesis [42] of *Eisenia fetida* showed that GABA-IR elements were primarily involved in the development and regeneration of the nervous system.

*Pygospio elegans* is a tube-dwelling annelid from the family Spionidae. Worms from this annelid family are widely distributed in intertidal sand flats in bays and estuaries of the Arctic Ocean and northern regions of the Pacific and the Atlantic [43, 44]. A small size and outstanding regenerative capacities make *P. elegans* a promising model for studies of reparative processes of annelids [44]. Based on morphological features and specific mode of segment regeneration of different body parts, *P. elegans* body segments can be subdivided into three regions [44–46]. The thorax consists of 12 segments without gills, however this number may vary from 10 to 13 in some cases. The abdomen consists of varying number of gill-bearing segments. The posteriormost “tail” region possesses segments without gills. The general morphology of anterior and posterior regeneration after traumatic amputation or asexual reproduction of *P. elegans* is well described [45, 46]. There are also some data on the structure and general course of nervous system regeneration visualized with antibody staining against acetylated α-tubulin, serotonin, and FMRFamide [47], as well as with histochemical staining for catecholamines [44].

Here we studied the nervous system of *Pygospio elegans* using commercially available antibodies against HA and GABA. Our aim was to broaden the repertoire of antibodies used to obtain new data for comparative analysis and to extend the knowledge about the morphology of the nervous system and stages of its regeneration in annelids. We also traced the reorganization of the nervous system during reparative regeneration in order to provide morphological background for further comparative morphological and molecular studies.

## Results

### Histamine-like immunoreactivity

**Fig. 1:**
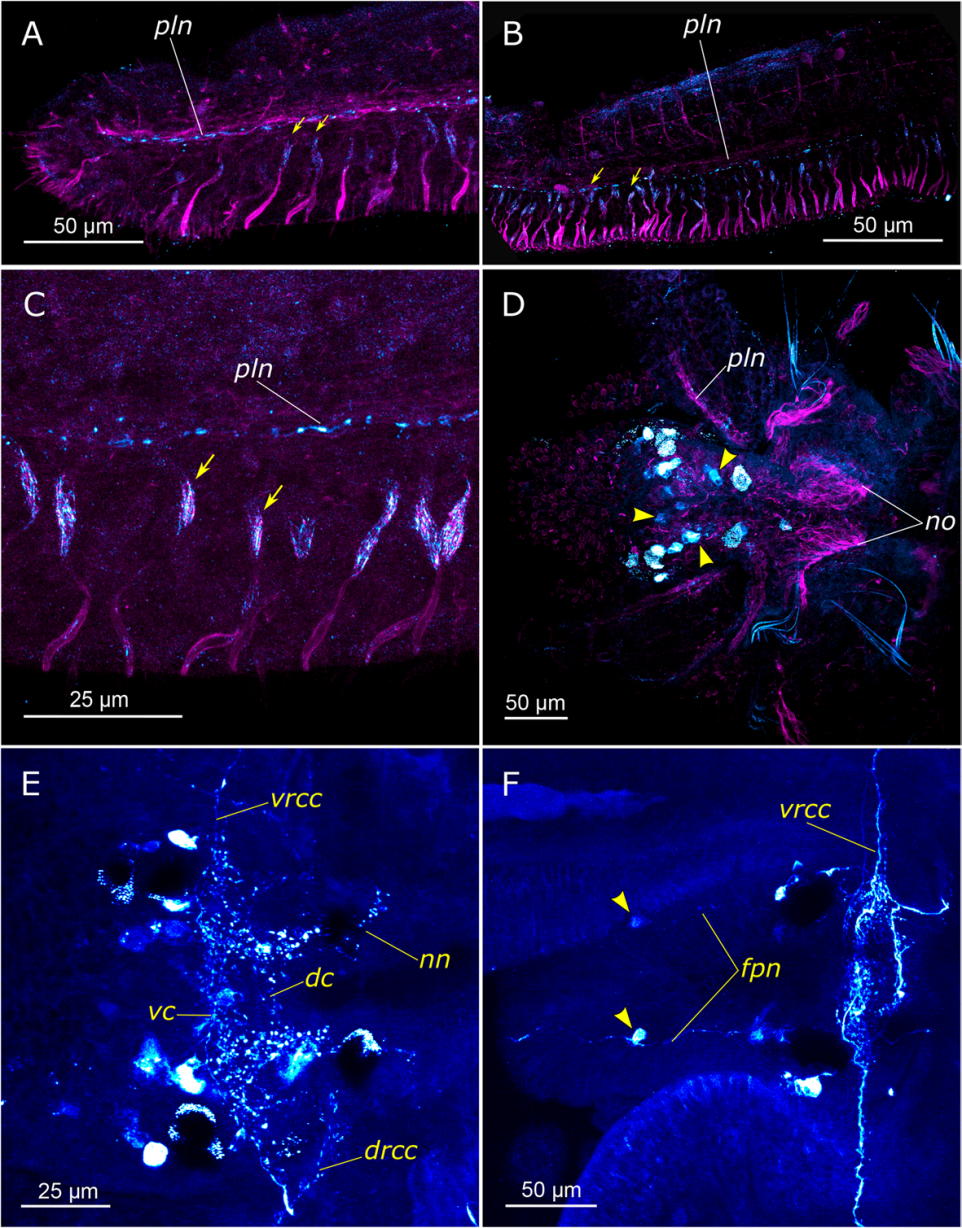
HA-like elements in palps and prostomium of *Pygospio elegans*. **a**,** b** HA-like elements in distal (**a**) and middle (**b**) parts of the palp. Yellow arrows label receptor cells. **c **High magnification of HA-positive palp receptor cells. **d – f** Partial z-projections through the cerebral ganglion in dorsoventral direction. HA-positive cell somata are labeled by yellow arrowheads. Abbreviations: *dc* – dorsal cerebral commissure, *drcc* – dorsal roots of the circumesophageal connectives, *fpn* – frontal posterior nerves, *nn* – nuchal nerve, *no* – nuchal organs, *pln* – palp nerve, *vc* – ventral cerebral commissure, *vrcc* – ventral root of circumesophageal commissure. HA-like elements – cyan to white, acetylated α-tubulin-like elements – magenta

HA-like immunoreactive elements were mostly found in the CNS. In the brain, about 30 HA-like neurons were located (Fig. 1d–f). Most of them contribute to a pair of cell clusters near the anterior eyespots. Two pairs of neurons were located closer to the rear pair of eyespots. The pigmented cups of the eyespots in most specimens show a bright autofluorescence and may thus be easily located. The fluorescent signal here is stronger than in neurons, and separate granules or vesicles are clearly distinguishable. The projections of HA-like immunopositive cells were found in the central neuropil in both dorsal and ventral commissures, ventral roots of the cerebral commissure, circumesophageal connectives,

stomatogastric nerves, nuchal nerves, palp nerves, and frontal prostomial nerves (Fig. 1a,b, e–f). A pair of cell somata was found in the anterior part of the prostomium (Fig. 1f). Their projections contribute to the frontal prostomial nerves.

Inside the palps HA-immunopositive elements were found in the main palp nerve, and could be traced to the very tip of the palp (Fig. 1a–b). Numerous regularly arranged HA-like immunopositive bipolar cells facing the food groove were located along the entire length of the palps (Fig. 1c). They possess a relatively thick dendrite, which is intensively labeled by anti-acetylated α-tubulin antibody.

**Fig. 2:**
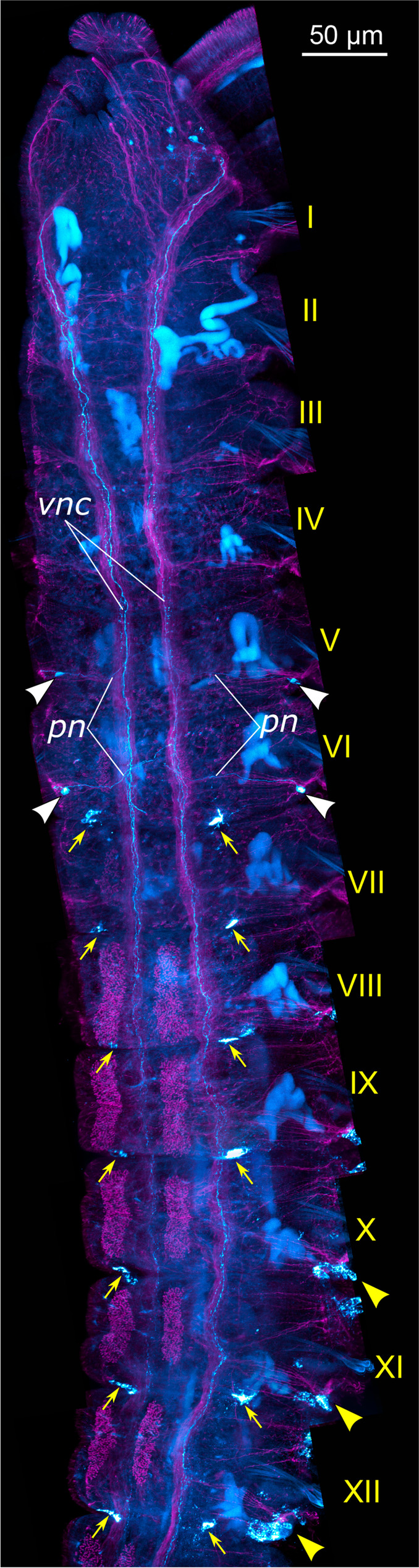
Panoramic micrograph showing general details of nervous system morphology and distribution of HA-like elements in thoracic segments of *Pygospio elegans* (ventral view). White arrowheads – HA-like peripheral neurons of thoracic segments, yellow arrowheads – HA-positive irregular shaped body wall cells, yellow arrows – ventral HA-like cell clusters. Roman numerals mark segmental numbers. Abbreviations**:** *pn* – parapodial nerve, *vnc* – ventral nerve cord. HA-like elements – cyan to white, acetylated α-tubulin-like elements – magenta

**Fig. 3:**
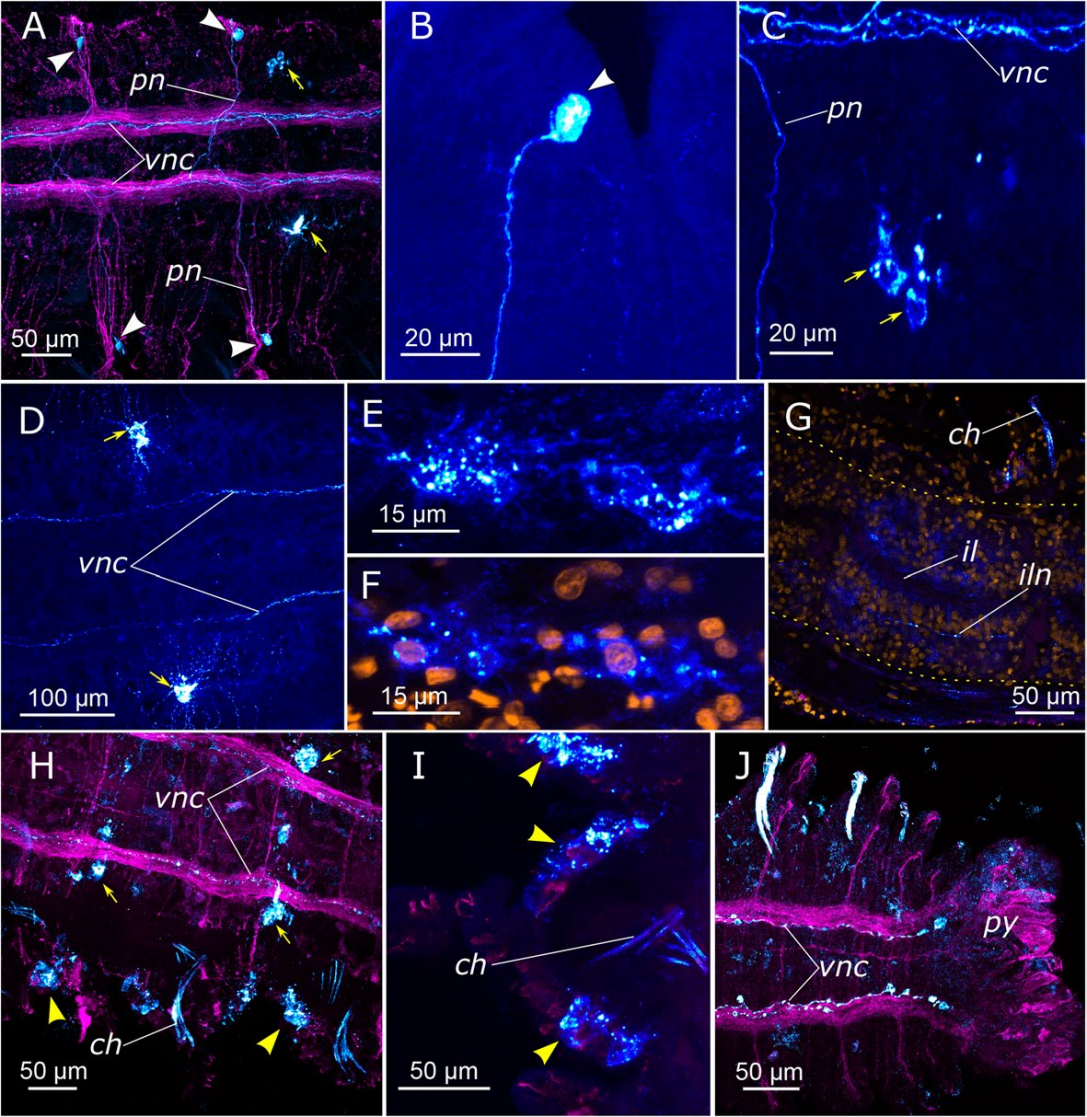
HA-like elements in body segments and the pygidium of *Pygospio elegans*. **a** Innervation of 5th and 6th thoracic segments. White arrowheads label the somata of HA-like peripheral neurons. Yellow arrows indicate HA-like cell clusters of the body wall. **b** Peripheral HA-like neuron of thoracic segment (white arrowhead). **c**,** d** Posterior thoracic segments with HA-like cell clusters in the body wall (yellow arrows). **e**,** f** High magnification of HA-like cells in the body wall. **g** Optical section through the body segments showing the innervation of intestine. Dotted lines outline the intestine. **h** Innervation of abdominal segments (ventral view). Yellow arrowheads label HA-positive irregularly shaped body wall cells. **i** Optical section showing the distribution of HA-like cells in the body wall of abdominal segments (yellow arrowheads). **j** Innervation of the pygidium and posterior segments (ventral view). Abbreviations: *ch* – chaetae, *il* – intestinal lumen, *iln* – intestinal longitudinal nerve, *pn* – parapodial nerve, *py* – pygidium, *vnc* – ventral nerve cord. HA-like elements – cyan to white, acetylated α-tubulin-like elements – magenta, cell nuclei – orange

In body segments, HA-like immunopositive elements were found mainly in the longitudinal nerves of the ventral nerve cord (Figs. 2 and 3a, h, j). In the abdominal and “tail” segments we did not find any other signal in CNS besides these nerves (Fig. 3h, j). HA-like fibers were located mainly in the paramedian nerves. Numerous varicose thickenings were seen in the region of the segmental ganglia neuropil. The HA-like immunopositive cell somata were not found in segmental ganglia.

In thoracic segments, the distribution of HA-like elements was unequal. A pair of neurons was found at the posterior ventral side of 5th and 6th thoracic segments at the bases of the parapodia (Figs. 2 and 3a, b). The cells are unipolar, sending their projections to the segmental nerves. They could be traced to the contralateral longitudinal nerves of the ventral nerve cord, thus making a chiasm.

Starting from the 6th thoracic segment, paired HA-like cell clusters were located at the ventral side of the worm along the posterior segmental border closer to the ventral nerve cord (Fig. 2 and 3c–f). The clusters contained 2–5 cells of irregular shape with numerous thin processes and a dense HA-like immunoreactive meshwork around them. There are also some HA-like immunoreactive cells of irregular shape in the body wall of abdominal and posterior thoracic segments. They are located laterally, closer to the margins of the segments (Fig. 3i).

HA-like immunopositive elements were not found in the pygidial region (Fig. 3j). In most specimens, paired HA-positive fibers ended with large vesicles at the border between the body segments and the pygidium. HA-like immunopositive signal was also detected in the longitudinal nerves supplying the intestine, though its intensity was relatively low (Fig. 3g).

### GABA-like immunoreactivity

**Fig. 4:**
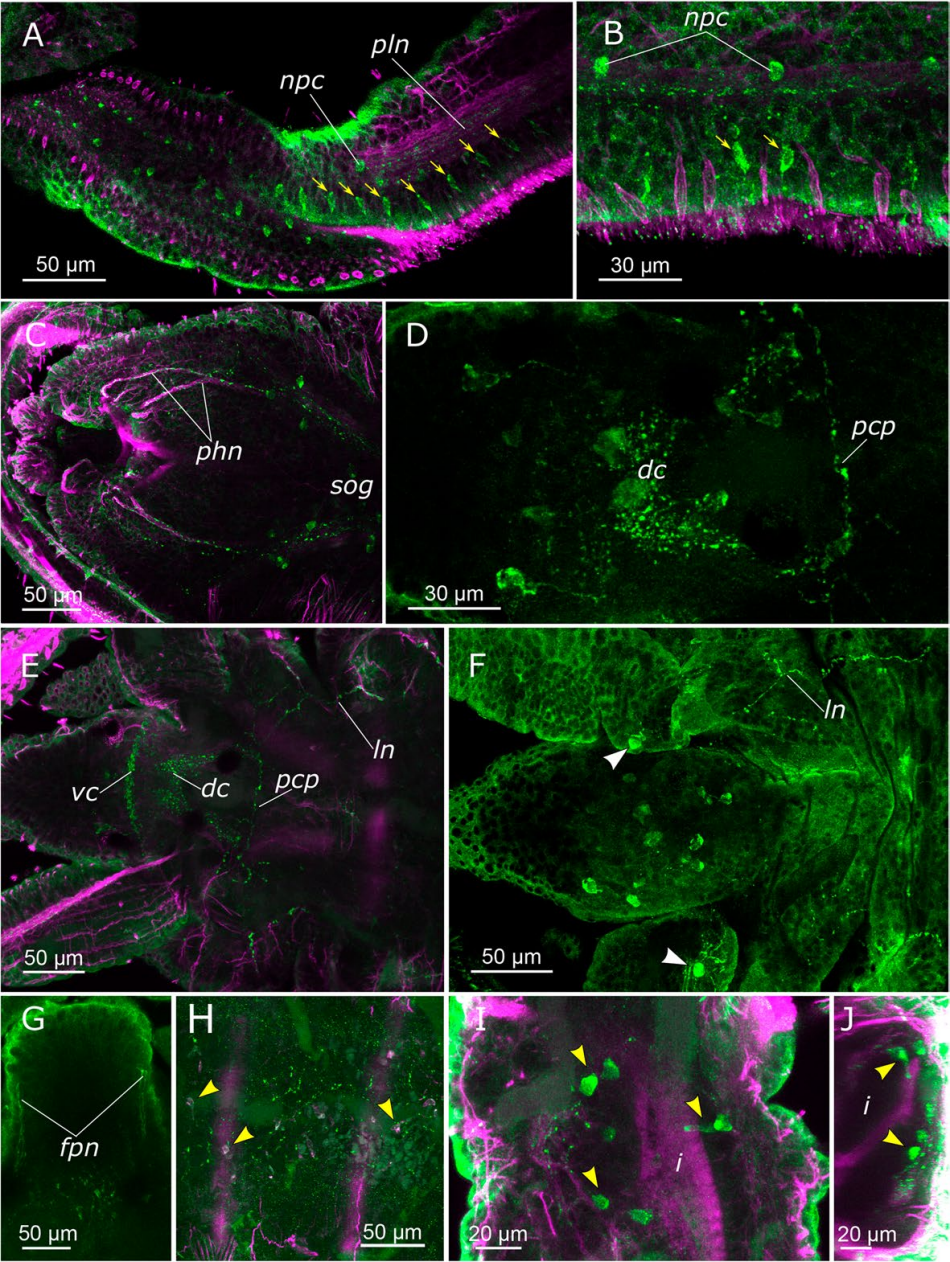
GABA-like elements in prostomium, palps and anterior body part of *Pygospio elegans*. **a**,** b** GABA-like elements of the palps. Yellow arrows label palp receptor cells. **c** Innervation of the mouth region, ventral view. **d-f** Partial z-projections of the brain region showing GABA-like neuronal somata (**d**,** f**) and fibers of GABA-like cells in the neuropil (**e**). White arrowheads – neurons at the palp base. **g** Innervation of the anterior part of the prostomium. **h – j** GABA-like elements in stomatogastric system of an intact (**h**) and regenerated specimens (**i**,** j**), 7 days after amputation (**i** lateral view, **j** virtual cross section through the thoracic segment). Yellow arrowheads mark neurons of the stomatogastric system. Abbreviations: *dc* – dorsal cerebral commissure, *fpn* – frontal prostomial nerve, *i* – intestine, *ln* – lateral longitudinal nerve, *npc* – non-receptor palp nerve cell, *pcp* – posterior commissure of the prostomium, *phn* – pharyngeal nerves, *pln* – palp nerve, *sog* – subesophageal ganglion, *vc* – ventral cerebral commissure. GABA-like elements – green, acetylated α-tubulin-like elements – magenta

**Fig. 5:**
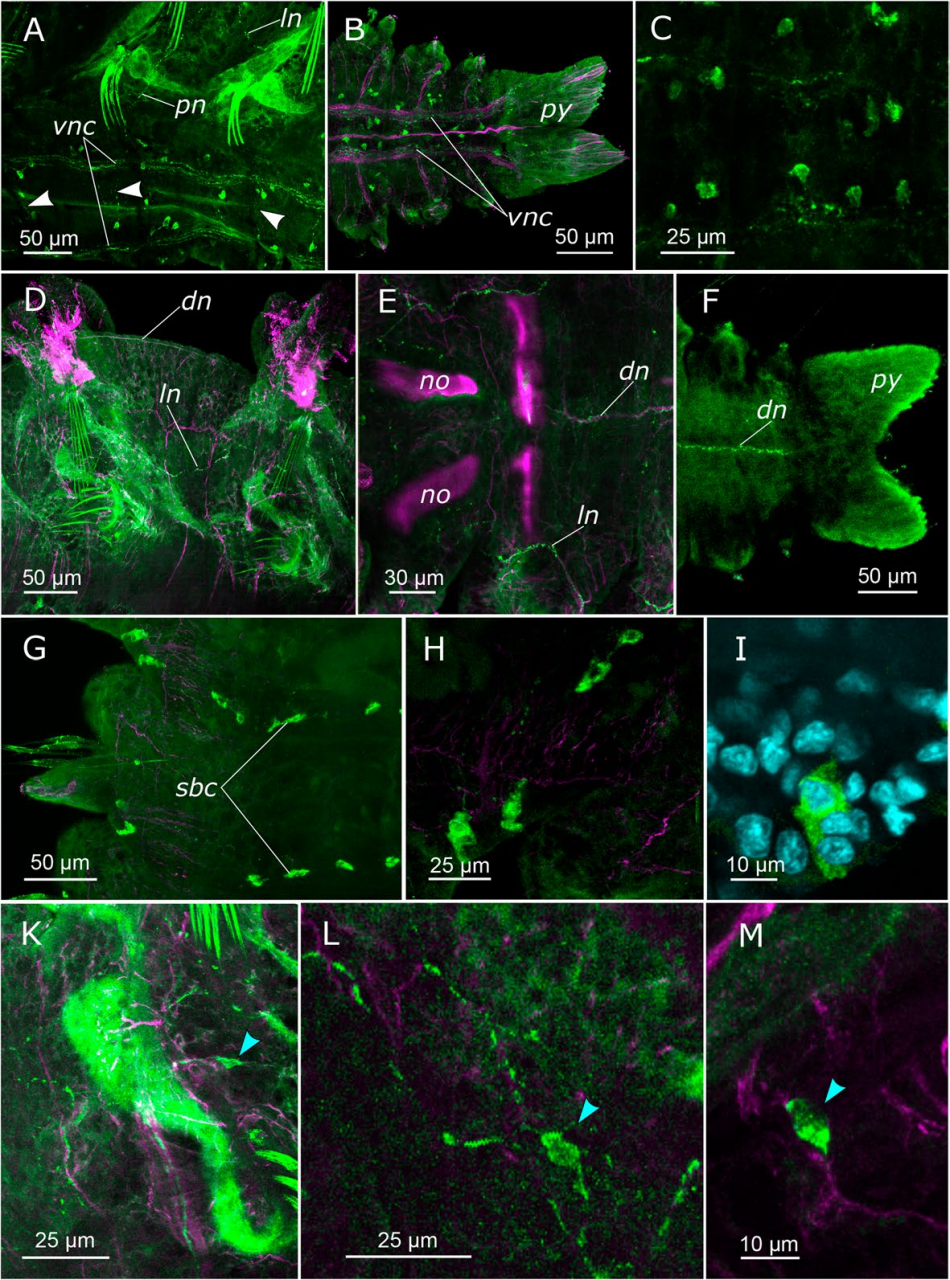
GABA-like elements in body segments and the pygidium of *Pygospio elegans*. **a**,** b** Innervation of abdominal segments (**a**) and pygidium (**b**), ventral view. White arrowheads label commissures of segmental ganglia. **c** GABA-like neuronal somata of the segmental ganglion. **d** Lateral longitudinal nerve in thoracic segments, lateral view. **e** Innervation of the second body segment, dorsal view.** f** Innervation of last abdominal segments and pygidium, dorsal view. **g-i** GABA-like bipolar cells in the body wall at the dorsal side of the segment. **k-m** Innervation of parapodia. Blue arrowheads – parapodial neurons. Abbreviations: *dn* – dorsal longitudinal nerve, *ln* – lateral longitudinal nerve, *no* – nuchal organ, *pn* – parapodial nerve, *py* – pygidium, *sbc* – segmental bipolar cells, *vnc* – ventral nerve cord. GABA-like elements – green, acetylated α-tubulin-like elements – magenta, cell nuclei – cyan

The used GABA antiserum gives nonspecific staining of the body surface, which, however, does not interfere with the labeling of nerve cells and their projections. The GABAergic system of *P. elegans* is well developed. It is represented by numerous fibers and somata both in the central and in the peripheral parts of the nervous system (Figs. 4 and 5). In the brain, the GABA antibody revealed approximately 20 cell somata and numerous fibers in the central neuropil. The cell somata do not form any distinct clusters in the brain, and their distribution is bilaterally symmetrical (Fig. 4d, f). They project axons to the ventral and the dorsal brain commissures, which are extensively labeled by GABA antibody. The fibers in the ventral commissure look like a nerve tract connecting the ventral roots of circumesophageal connectives. It also gives rise to paired frontal prostomial nerves (Fig. 4g). The dorsal commissure is thicker than the ventral one and has a butterfly-like shape. The fibers projected from its posterior parts are connected with the posterior commissure of the prostomium, which is situated between the posterior pair of eyespots and the nuchal organs (Fig. 4d, e). The posterior commissure of the prostomium gives rise to longitudinal lateral peripheral nerves (Fig. 4e, f). Numerous GABA-like immunopositive elements were found along the palps.

A pair of intensively labeled cell somata was found in the palp bases. The cells are unipolar and lie closer to the main palp nerves. Numerous immunoreactive fibers contribute to the main palp nerves. In the additional palp nerves the immunoreactive signal was not detected (Fig. 4a, b). Two rows of bipolar GABA-like immunopositive cells were located along the food groove (Fig. 4a). Their projections face the body surface inside the food groove closer to the midline. A strong fluorescence of cilia in the food groove makes it impossible to determine whether these cells have their own cilia. In addition, a row of regularly spaced GABA-positive cells was found along the main palp nerve (Fig. 4b, *npc*). The cells do not reach the epithelial surface and are located in the inner sides of the palps.

The nerve plexus, which constitutes the stomatogastric system, was also labeled by GABA antibody (Fig. 4h–j). It is more conspicuous in regenerating worms, which have a thinner body wall facilitating the visualization of the internal structures. Most cells contributing to the plexus are bipolar cells with rounded somata. There are also pyriform cells with short processes facing the intestinal lumen.

The subesophageal connectives show a strong GABA-like immunopositive fluorescence. On the ventral side they give rise to the paired pharyngeal nerves. They branch further, innervating the surface of the lower lip from the ventral side and then going to the pharynx (Fig. 4c). The subesophageal ganglion has a strong GABA-positive innervation. It contains a number of cells and has a thick commissure, which was not detected in the ganglia of the following segments. In subsequent body segments GABA-like immunopositive elements were found mostly in the ventral nerve сord (Fig. 5a–c). Up to 14 neuronal somata can be detected in each ganglion. Their projections form a well-developed neuropil. However, the GABA-positive commissures were relatively thin and contained only single fibers (Fig. 5a). They were situated at the posterior part of the ganglion.

Fibers of GABA-positive cells were found in three peripheral longitudinal nerves. A pair of lateral longitudinal nerves originates in the posterior part of the prostomium and run dorso-laterally in the body segments above the parapodia (Fig. 5a, d, e). An unpaired dorsal nerve runs along the dorsal midline and can be traced from the second thoracic segment to the pygidium (Fig. 5d–f).

Fibers of GABA-like immunopositive cells were detected in two segmental nerves. The first one is located in the anterior part of the segment. It usually had a faint fluorescent signal. The second GABA-positive segmental nerve supplies the parapodium (Fig. 5a). It goes along its posterior side, crosses the lateral longitudinal nerve and proceeds to the dorsal side connecting to the dorsal unpaired longitudinal nerve (Fig. 5d–f).

At the dorsal side of the body closer to the posterior segment boundary, the GABA antibody labeled a row of bipolar cells (Fig. 5g–i). They are spindle-shaped and have thick short processes facing the body surface. Bipolar GABA-like immunopositive cells were found in the body wall at the bases of the parapodia (Fig. 5k–m). They project fibers to the plexus of the body wall.

GABA-like immunopositive fibers enter the pygidial longitudinal nerves as parts of the ventral nerve cords. However, we did not find any other GABA-like elements in this body region (Fig. 5b, f).

**Fig. 6:**
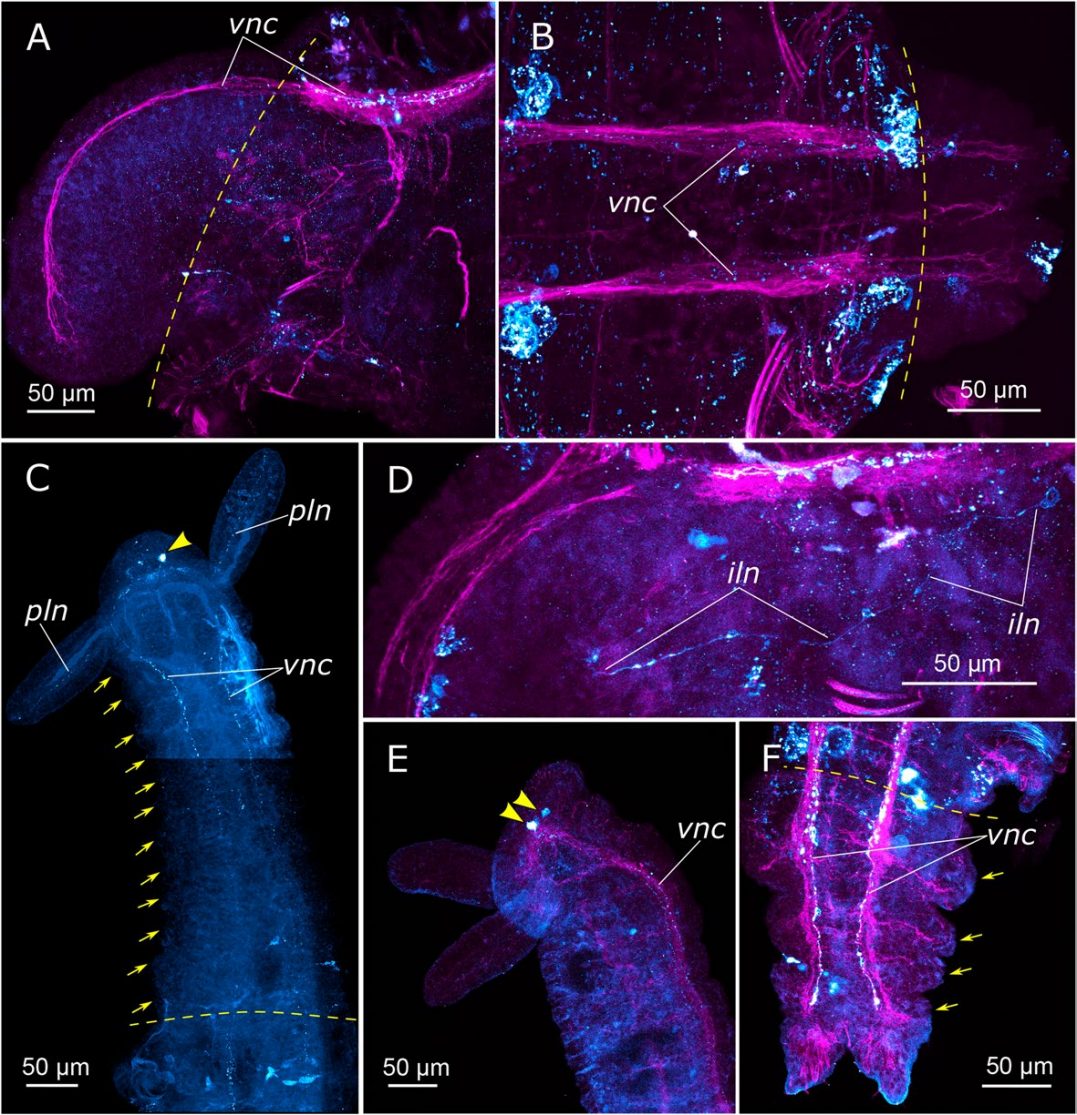
Regeneration of the HA-like elements of *Pygospio elegans* nervous system. **a** Growing head, 3 dpa (lateral view). **b** Growing “tail”, 4 dpa (ventral view). **c** Growing head and thoracic segments, 7 dpa (ventral view). Yellow arrowhead label the first HA-positive neurons, yellow arrows – newly grown thoracic segments. **d** Growing head, 4 dpa (lateral view). **e** Growing head, 7 dpa (lateral view). Yellow arrowheads – the first HA-positive neurons. **f** Growing “tail”, 7 dpa (ventral view). Yellow arrows – newly grown segments. Yellow dashed line marks the cut line. Abbreviations: *iln* – intestinal longitudinal nerve, *pln* – palp nerve, *vnc* – ventral nerve cord. HA-like elements – cyan to white, acetylated α-tubulin-like elements – magenta

### Regeneration of HAergic system

At early stages of regeneration (0–48 hours post amputation) we did not find any signs of reorganization in HAergic system in both anterior and posterior regenerates. HA-like immunofluorescent elements could be detected in the area of the anterior regenerate for the first time after 3 days post amputation (dpa) (Fig. 6a, d). These elements were represented by fibers contributing to the longitudinal nerves of the ventral nerve cord (VNC), stretching from the first intact segmental ganglion. By that time HA-like fibers did not reach the prostomium yet. The fibers of the intestinal nerves also entered the regenerate at 3 dpa. Surprisingly, we did not find any accompanying anti-acetylated α-tubulin signal in the intestinal nerve, either in intact or in regenerating worms (Fig. 6d). HA-like immunopositive fibers outlined all the main nerves and tracts of the prostomium by 7 dpa (Fig. 6c). First one or two pairs of cells appear in the brain (Fig. 6c, e). The first HA-positive elements in the palps also become visible after 7 dpa (Fig. 6c).

In the posterior regenerate, HA-like immunopositive fibers started to grow only at 3 or even 4 dpa (Fig. 6b). At 7 dpa, the HAergic system of the posterior end of the regenerate was fully reorganized, although the border between the intact and the regenerated part was still clearly visible (Fig. 6f).

### Regeneration of GABAergic system

**Fig. 7:**
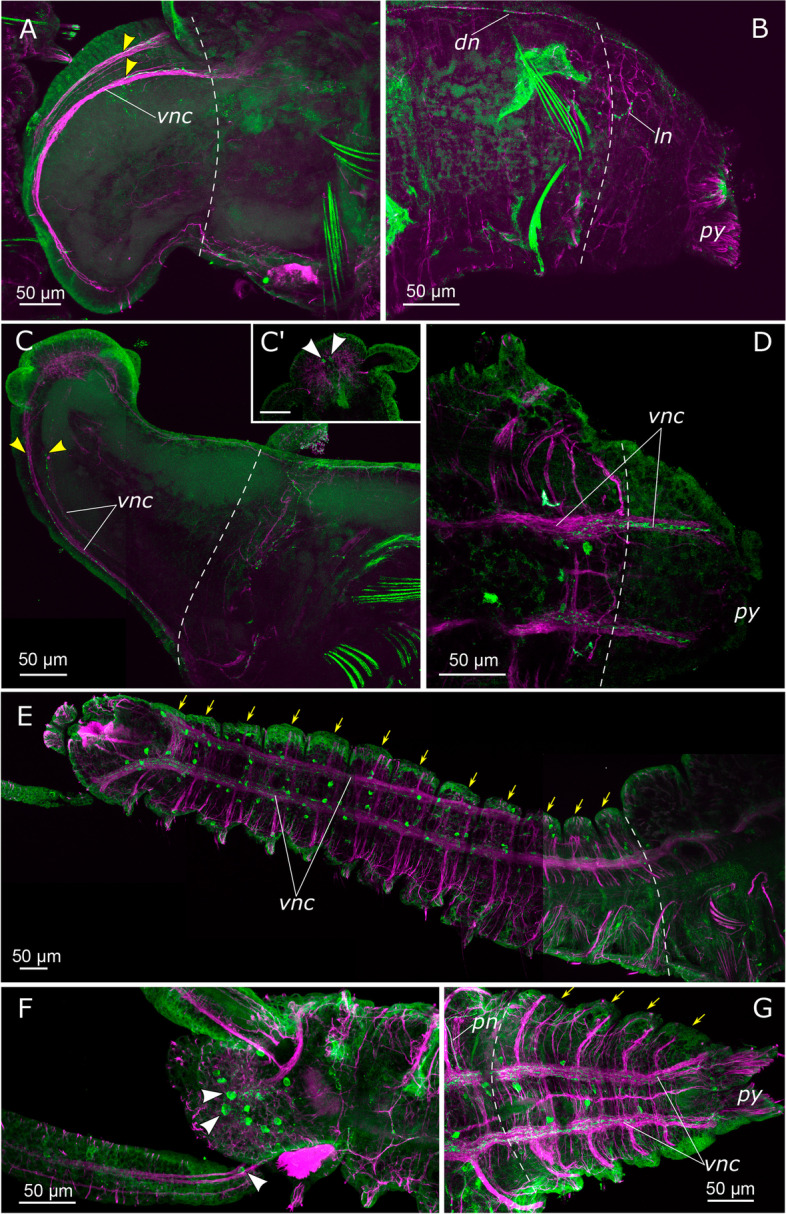
Regeneration of the GABA-like elements of *Pygospio elegans* nervous system. **a** Growing head, 3 dpa (lateral view). Yellow arrowheads – GABA-like nervous fibers. **b** Growing “tail”, 3 dpa (lateral view). **c, c’**. Growing head, 4 dpa (**c** – dorsolateral view, **c’** - dorsal view). Yellow arrowheads - GABA-like nervous fibers, white arrowheads – first GABA-positive neurons. **d** Growing “tail”, 4 dpa (ventral view). **e** Growing head and thoracic segments, 7 dpa (ventral view). Yellow arrows – newly grown thoracic segments. **f** Growing head, 7 dpa (dorsal view). White arrowheads – GABA-positive neurons. **g** Growing “tail”, 7 dpa (ventral view). Yellow arrows – newly grown segments. White dashed line marks the cut line. GABA-like elements – green, acetylated α-tubulin-like elements – magenta. Abbreviations: *dn* – dorsal longitudinal nerve, *ln* – lateral longitudinal nerve, *py* – pygidium, *vnc* – ventral nerve cord

The first signs of reparation of GABA-like immunopositive elements in both the anterior and the posterior regenerate become visible at 3 dpa. In the anterior regenerate, the longitudinal fibers of the VNC starting from the first intact segment were the first to appear (Fig. 7a). At 4 dpa, GABA-positive fibers could be traced up to the head lobe (Fig. 7c), and the first cells (2–4 neurons) appeared in the brain (Fig. 7c’), though their fluorescence was faint and their projections were almost impossible to trace. After that, an active differentiation of neurons in the newly formed segmental ganglia and in the brain began. The process was very fast, so that by 7 dpa almost all the neurons, including the neuronal elements of the palps, were differentiated (Fig. 7e, f).

In the posterior regenerate, the difference between 3 dpa and 4 dpa was almost indiscernible. At the ventral side, GABA-positive fibers could be detected as a part of the newly formed longitudinal nerves of the VNC going down to the pygidium (Fig. 7b, d). Besides the VNC, GABA-like immunopositive fibers start to grow into the posterior regenerate from dorsal and lateral longitudinal nerves (Fig. 7b). By 7 dpa, GABAergic system of the posterior regenerate did not differ from that of the intact segments (Fig. 7g). However, the fibers in the commissure and segmental nerves were not clearly distinguishable in newly formed segments.

## Discussion

**Fig. 8:**
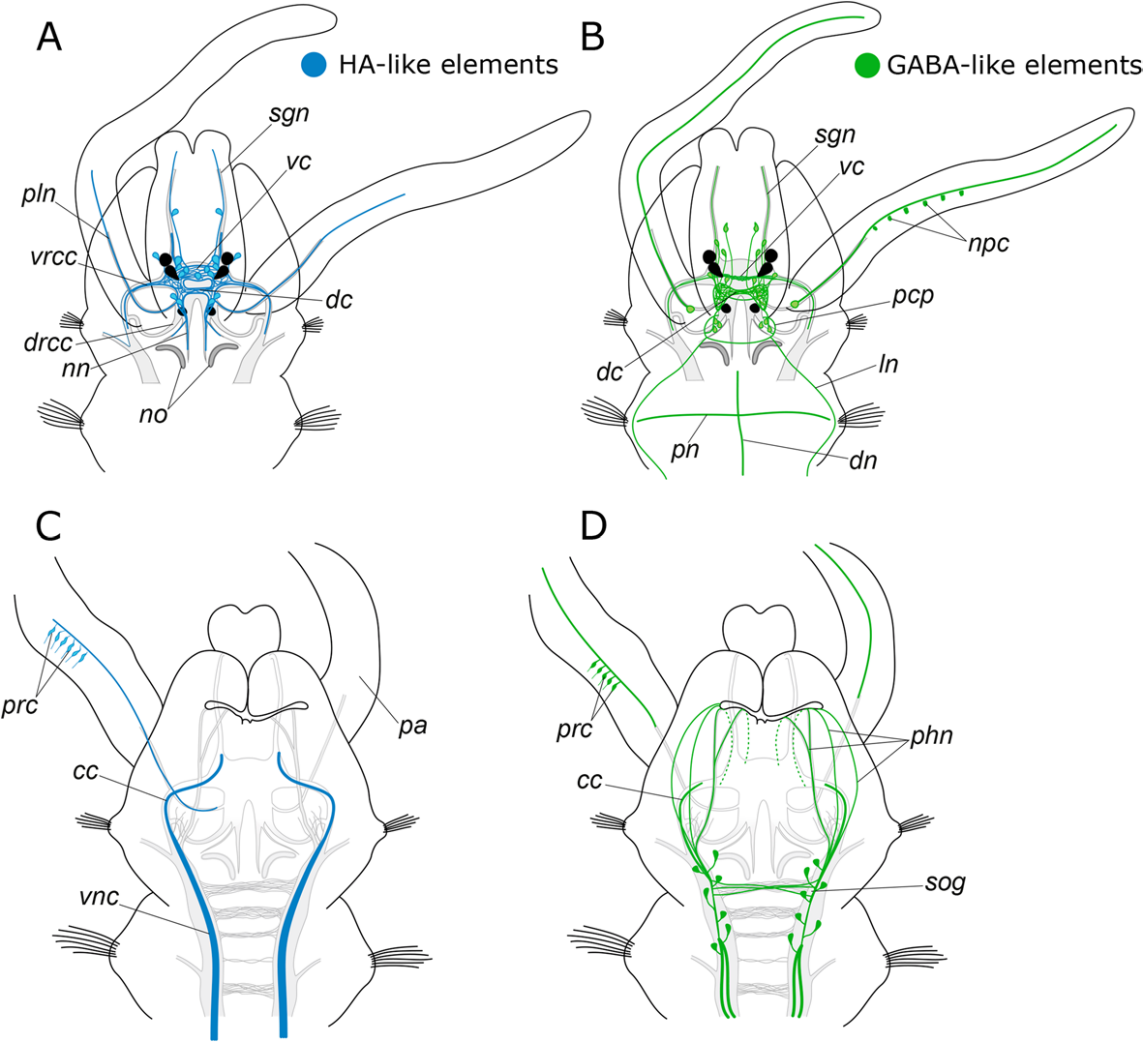
Schematic representation of HA- and GABA-like elements in the anterior part of *Pygospio elegans* nervous system. The main nerve tracts of the central nervous system are outlined in grey. **a** HA-like elements (dorsal view). **b** GABA-like elements (dorsal view). **c** HA-like elements (ventral view). **d** GABA-like elements (ventral view). Abbreviations: *cc* – circumesophageal connectives, *dc* – dorsal cerebral commissure, *drcc* – dorsal roots of the circumesophageal connectives, *dn* – dorsal longitudinal nerve, *ln* – lateral longitudinal nerve, *nn* – nuchal nerve, *no* – nuchal organs, *npc* – non-receptor palp nerve cells, *pa* – palps, *pcp* – posterior commissure of the prostomium, *phn* – pharyngeal nerves, *pln* – palp nerve, *pn* – parapodial nerve, *prc* – palp receptor cells, *sgn* – stomatogastric nerve, *sog* – subesophageal ganglion, *vc* – ventral cerebral commissure, *vnc* – ventral nerve cord, *vrcc* – ventral root of circumesophageal connectives

**Fig. 9:**
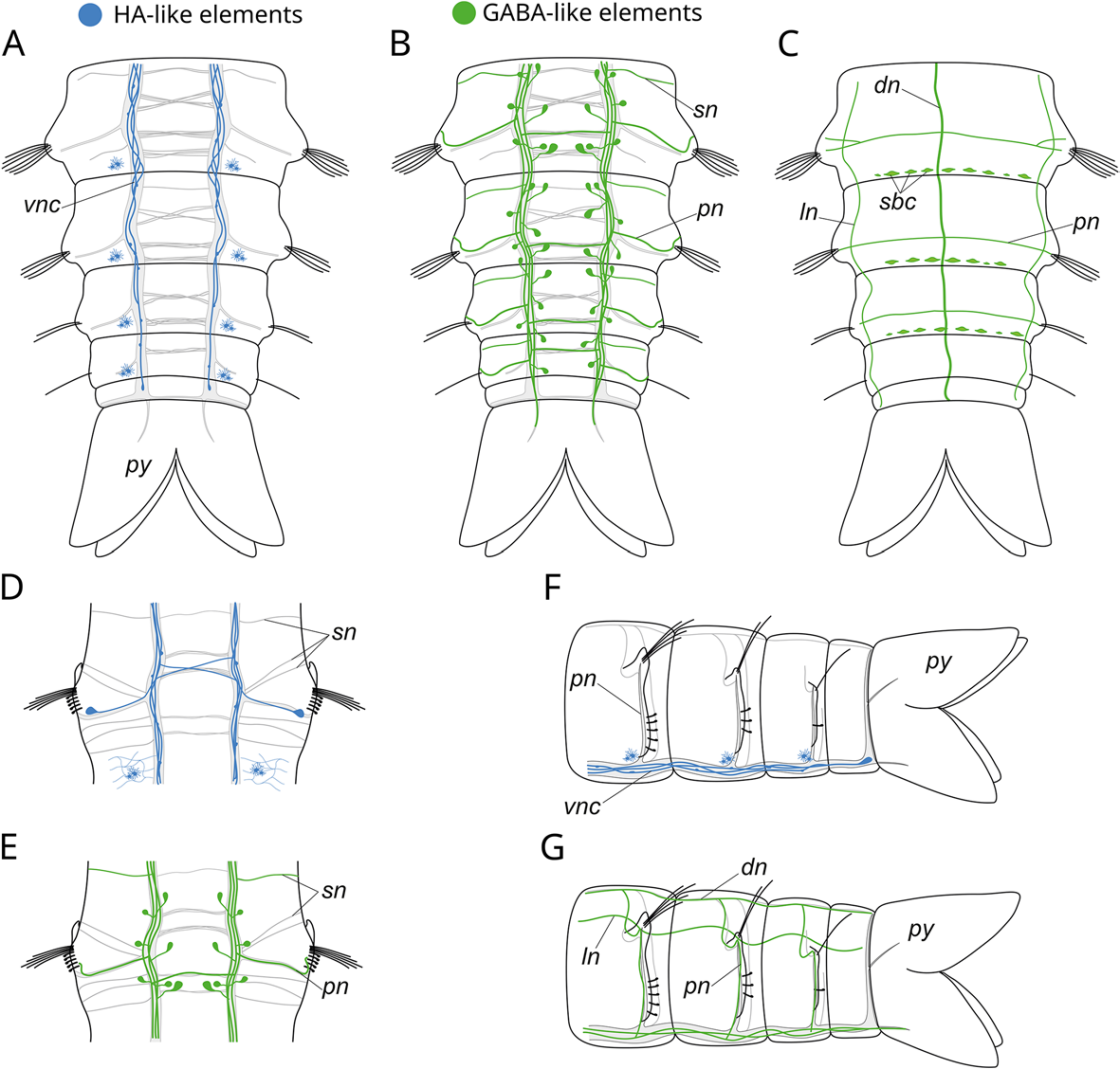
Schematic representation of HA- and GABA-like elements in body segments and pygidium of *Pygospio elegans*. The main nerve tracts of the central nervous system are outlined in grey. **a** HA-like elements of posterior body segments (ventral view). **b**,** c** GABA-like elements of posterior body segments, ventral (**b**) and dorsal **(c)** views. **d** 5–6th thoracic segment HA-like elements (ventral view). **e** GABA-like elements of thoracic segment (ventral view). **f**,** g** HA-like (**f**) and GABA-like (**g**) elements in the posterior body segments (lateral view). Abbreviations: *dn* – dorsal nerve, *ln* – lateral nerve, *pn* – parapodial nerve, *py* – pygidium, *sbc* – segmental bipolar cells, *sn* – segmental nerve, *vnc* – ventral nerve cord

In this study we mapped the distribution of histamine and gamma-aminobutyric acid in the nervous system of *P. elegans* using commercially available antibodies. The schematic representations of their distribution are presented in Figs. 8 and 9. We also described the distribution of these neuroactive substances in the reparative regeneration of the nervous system. These two neurotransmitters had an unequal distribution in the central and the peripheral parts of the nervous system. Our results suggest that each type of the labeled cells has a specific, apparently non-overlapping localization in the central and the peripheral parts of the nervous system and that the specification of neurons and nerve fibers in the regenerates of *P. elegans* follows a specific order.

### Histamine

The most intriguing feature of HA distribution in CNS of *P. elegans* is an exclusive localization of the neuronal somata in the brain (Fig. 8a, c). VNC is devoid of cell somata and contains only fibers in longitudinal nerves (Fig. 9a, f). No segmental nerves contained HA-like immunoreactive fibers. Thus, we conclude that most of the HAergic cells in the CNS of *P. elegans* are interneurons performing integrative functions and coordinating inter-segmental neuronal activity. The only other annelid in which the distribution of HA in the CNS was described is *Nereis diversicolor* [32], though only its brain was studied. The distribution of HA-positive elements in the brain of *P. elegans* is broadly similar to that in the brain of *N. diversicolor*, indicating that HA-positive elements in these two species are generally homologous.

HA-like immunoreactivity was revealed in the main palp nerves, which is also in accordance with the data from *Nereis diversicolor* [32]. The antibody also labeled a number of bipolar cells with relatively thick and long processes facing the lateral sides of the food groove. Judging from their morphology and localization, these cells are probably sensory. This observation corresponds to the findings of HA-positive cells in clitellates [48]. The body wall epithelium of *Lumbricus terrestris* and *Eisenia fetida* was shown to contain bipolar HA-immunoreactive sensory cells as well as long processes coming from deeper regions of the body wall. Similar HA-immunoreactive sensory cells are characteristic of sense organs in mollusks [30, 49–51]. These observations support the idea that HA plays similar sensory functions in these two phyla.

Another interesting finding is the presence of paired unipolar HA-positive cells at the bases of parapodia in 5th and 6th thoracic segments (Fig. 9d). There are no data about the origin and functions of these neurons. They may be involved in reproduction, reception, or tube building. Further studies of these cells and related structures in various taxa are desirable.

Starting from 6th body segment, paired HA-positive cells with numerous processes can be seen in the ventral posterior parts of each segment. These cells may be recognized as fibroblasts or glial cells but not neurons. In vertebrates histamine-containing cells participate in different physiological functions such as the inflammatory response [52]. According to our observations, there is no specific involvement (degranulation or migration) of HA-positive non-neuronal cells in wound healing and further reparative processes in *P. elegans*. Further studies can clarify the nature of these cells and their functions.

### GABA

The identification of GABA-positive nerve elements was hindered by a strong non-specific antibody binding. A weak background signal registered in the body wall made it difficult to isolate some thin immunoreactive elements in the nervous system. Nevertheless, a careful slice-by-slice analysis made it possible to identify GABA-like immunopositive cells and their projections in CNS and peripheral nerves.

GABA was more widely distributed in *P. elegans* than histamine. The antibody labeling revealed numerous GABA-positive cells in the brain and segmental ganglia as well as in palps and the body wall (Fig. 8b, d; Fig. 9b, c). This finding is in excellent agreement with the results obtained on clitellates *Lumbricus terrestris* [53] and *Eisenia fetida* [48], although the number of labeled cells in *P. elegans* was significantly lower. In *L. terrestris* GABA-like cells form distinct clusters in the brain and ventral ganglia. In *P. elegans* the cell somata are distributed more or less evenly, which may be partly explained by the fact that there are fewer cells. It is also noteworthy that the GABA content in segmental nerves was relatively low (Fig. 9e, g). According to our data, *P. elegans* possesses at least 5 pairs of segmental nerves revealed by acetylated α-tubulin immunoreactivity (Fig. 4g), but only two of them demonstrate a weak GABA-like immunoreactivity. At the same time, GABA-like immunoreactive fibers are abundant in the neuropil. In *L. terrestris* many GABA-positive cells in the VNC were recognized as interneurons sending their projections either to the neuropil of the same ganglion or to the other ganglia, with only a few neurons being projected to the periphery [53]. This also seems to be the case in *P. elegans*. In mollusks GABA-like immunoreactive cells are also generally located in the CNS and their peripheral projections are scarce [54–56], indicating primary central action of GABAergic neurons [57]. This might indicate a conserved function of GABA-positive cells in annelids.

One of our most intriguing results is the discovery of GABA-like immunopositive cells along the palps. There are, apparently, two types of these cells (Fig. 8b, d). The cells of the first type are bipolar, projected to the surface with short thin processes, and lying in two rows inside the food groove. They can thus be regarded as sensory cells. GABA-positive sensory cells of a similar kind were found in the body wall of clitellates *Eisenia fetida* and *Lumbricus terrestris* [48]. Clitellates have no palps, so the sensory cells involved in feeding behavior should be located directly in the body wall of the anterior segments. This is corroborated by the experimental data: the number of sensory cells in body wall gradually decreased in antero-posterior direction [48]. Therefore, we suggest homology of GABA-positive sensory cells in spionids and clitellates, however, the exact functions of these cells are still unknown. In gastropod mollusks GABA-positive cells were also shown to be sensory, and their chemoreceptive functions were described [58, 59].

The cells of the second type, which are less numerous, are located at inner sides of the palps. Unlike the cells of the first type, they have no distinct projections to the surface and lie closer to the main palp nerve. We speculate that these cells are neurons that modulate the palp activity, but this needs further study. Moreover, their sensory nature cannot be ruled out, either, since the presence of sensory cilia on abfrontal sides of the palps in *P. elegans* has been shown [60]. In the osphradium of a gastropod *Lymnaea stagnalis* the colocalization of GABA and FMRFamide was demonstrated in ganglion neurons and in sensory cells penetrating the epithelium [58]. In *P. elegans* FMRFamide-like immunoreactive cells were also found in the palps [60], but their localization differed from that of the GABA-like cells found in this study.

A number of GABA-like cells was also found in the stomatogastric nervous system. Similar cells reported for *E. fetida* and *L. terrestris* [53, 61] were suggested to have, based on their localization and morphology, sensory as well as endocrine function. GABA-positive cells in the stomatogastric system in *P. elegans* found in our study may have similar functions. However, all these conclusions are based on indirect evidence, and further studies are needed to discover the exact functions of GABA in stomatogastric system of annelids. The results of studies of the role of GABA in the regulation of the muscular activity in the body wall of annelids are contradictory. In a clitellate *Pheretima communissima*, both inhibitory and excitatory effects of GABA on spontaneous muscle contraction have been detected [62, 63]. In another clitellate, *Eisenia fetida*, GABA had no effect on muscle contraction in the body wall but could inhibit serotonin-mediated contractions [48]. GABA-like immunopositive fibers in the wall of body segments of *P. elegans* are restricted to a few longitudinal and transversal nerves running along the main muscular bundles, which suggests the involvement of GABA in neuromuscular transmission. The fact that GABA-immunoreactive fibers were also observed in the body wall musculature of *L. terrestris* [53] indicates a possible common function of GABA in annelids*.*

### Regeneration

The overall pattern of the nervous system regeneration recorded in our study is similar to those described for other annelids [8, 12, 47, 64]. The redevelopment of the posterior end of the body is in general similar to that of the anterior one but is faster. The difference in the speed of this process is explained by its different mode in the anterior and the posterior body end. The posterior regeneration is restricted to the reconstitution of the pygidium and the growth zone, while the anterior one requires the redevelopment of all the lost thoracic segments as well as the prostomium [44].

The difference in the nervous system regeneration in different body parts reflects the overall difference in anterior and posterior regeneration. Nevertheless, in both the anterior and the posterior regenerates the neuroactive substances examined in our study became detectable starting from 3 dpa (stage 3 according to [44]). At this stage the nerve fibers have already infiltrated into the regenerate and the scaffold of the regenerating CNS is recognizable. This is in contrast to the studies of serotoninergic system, whose nerve fibers were detected in regenerating parts at the first steps of the nervous system redevelopment [8, 64, 65]. In *P. elegans* the nervous system regeneration was previously studied by Lindsay et al. [47]. The authors showed that at 3 dpa post-ablation FMRFamide-immunoreactive elements were already detected in the brain in *P. elegans* and *Dipolydora quadrilobata*. However, no data about earlier stages were presented and the experiments were performed at lower temperatures than in our study, making direct comparison problematic. Our preliminary data on serotonin- and FMRFamidergic systems regeneration support the results by Lindsay et al. [47] and demonstrate that these two neurotransmitters appear in the area of both the anterior and the posterior regenerates starting from the very first steps of the nervous system redevelopment (data unpublished). This means that the specification of neurons with different chemism in the course of regeneration is not simultaneous.

Further steps of the nervous system regeneration in *P. elegans* are characterized by the unequal rates of redevelopment of different neurotransmitters. HA-positive neuronal somata in the brain become visible only at 7 dpa, while for GABA this process is much faster: the first neuronal somata are detectable at 4 dpa, and by 7 dpa almost all GABAergic system is redeveloped. We have previously studied the regeneration of catecholaminergic (CA) system of *P. elegans* using glyoxylic acid induced fluorescence method [44]. The first CA-positive elements in the regenerates were detected staring from 2 dpa. After 4 dpa the regeneration speeds up, and by 7 dpa the reparation of both the central and the peripheral parts of the nervous system are almost completed. This dynamics resembles that of GABAergic system regeneration, though the reconstitution of the peripheral nervous system begins prior to that of the CNS. Thus, the time needed for the full redevelopment of different neuroactive substances is different. This difference may correlate with neuronal development during embryogenesis. In most of the studies involving serotonin and FMRF-amide-positive nervous systems there was a clear sequence of NS development [66–68]. In clitellates, for example, GABA-positive cells were reported before serotonin-positive ones [42, 69], and this observation was corroborated by the data on brain regeneration [41, 70].

## Conclusions

Data on the content and distribution of neurotransmitters are valuable for comparative analysis, and may act as a bridge between form and function. Here we provided new data on the nervous system organization in spionid annelids and the distribution of HA- and GABA-immunopositive cells in it. Comparison with clitellates and mollusks demonstrated a general similarity in HA and GABA distribution, which may be a result of common basic functions of these neurotransmitters in the Trochozoa. At the same time, we found numerous unique features in HA and GABA distribution in *P. elegans*. This finding indicates an evolutionary plasticity of the nervous system. We also found that HA- and GABA-immunopositive receptor cells were well-represented in palps of *P. elegans*, suggesting a high complexity of these sensory organs and a high diversity of chemical specificity of sensory cells. The sequential steps identified in the process of the nervous system regeneration provide new insights on correlation between reparative and developmental processes. The difference between the full redevelopment of various neurotransmitters correlates with neuronal development during embryogenesis.

Taken together, our results illustrate the importance of taking into account the distribution of various neurotransmitters in comparative morphological and developmental studies. Unfortunately, there are almost no data on the distribution, development and regeneration of HAergic and GABAergic cells in the nervous system of members of other annelid families. Further comparative studies of various neuroactive substances in different annelid clades are needed.

## Methods

### Animal collection and keeping

Worms *Pygospio elegans* Claparède, 1863 were collected at low tide from mud and sand flats near the marine biological station Dalnie Zelentsi (69°07′ N, 36°05′ E) in the Barents Sea region. After collection, the worms were placed in plastic containers with prepared sieved sand (≥0.5 mm). They were transported to the laboratory and kept there at 18 °C in the same containers. Artificial seawater (Red Sea Coral Pro salt, Israel) with a salinity of about 30–32‰ was used. It was changed regularly, and the worms were fed on powdered algae collected from the same intertidal zone. Under these conditions, the worms could be successfully maintained for several months.

### Sampling, experiments, fixation

For regeneration experiments, the worms were dissected with razor blades after the 20th body segment. After that, both halves were kept in separate Petri dishes filled with artificial seawater at 18 °C. To describe the entire regeneration process, we used the following fixation points: 0 hours post-amputation (hpa), 4 hpa, 10 hpa, 18 hpa, 24 hpa, 48 hpa, 3 days post-amputation (dpa), 4 dpa, 7 dpa.

All animals were relaxed in a 7.5% solution of MgCl_2_• 6H_2_O before fixation. Two different fixatives were used for immunochemistry: freshly prepared 3.7% paraformaldehyde +0.3% glutaraldehyde on 0.1 M phosphate-buffered saline (PBS) for staining with GABA antibodies and 4% 1-ethyl-3(3-dimethylaminopropyl)-carbodiimide following with the postfixation with 4% paraformaldehyde for labeling with histamine antibody [32]. Totally, about 10 worms were fixed for each type of antibody at each time point of regeneration. After fixation, the samples were washed 3x20 min in PBS, and then kept in 1x PBS containing 0.03% sodium azide at 4 °C.

### Immunochemistry

Before staining, the worms were rinsed in PBS and preincubated overnight in PBS with 0.25% bovine serum albumin (BSA). The following primary antibodies were used: polyclonal rabbit anti-histamine (Immunostar, 22,939) and polyclonal rabbit anti-GABA (Immunostar, 20,094). The primary antibodies were diluted 1:100 in PBS with 0.25% BSA. To label the overall nerve scaffold the monoclonal mouse anti-acetylated α-tubulin antibody (Sigma, T-6793) was added in dilution 1:2000. The specimens were incubated in primary antibodies for 16-24 h at 4 °C, then washed 3x20 min in PBS and incubated with secondary antibodies Alexa Fluor 488 Donkey Anti-Rabbit (Molecular probes, A-21206) and Alexa Fluor 647 Donkey Anti-Mouse (Molecular probes, A-31571) both diluted 1:500–1:1000 overnight at 4 °C. Then the specimens were again washed several times in PBS, mounted in Mowiol 4–88 with DAPI (Carl Roth, 6335.1) between two coverslips and stored at 4 °C for 1–7 days before microscopy.

### Microscopy and image processing

The specimens were examined using a laser-scanning microscope Leica TCS SP5 (Leica Microsystems, Wetzlar, Germany). The specimens were scanned in 50–100 optical sections with a thickness of 0.5 μm and then processed with Fiji [71] and Bitplane IMARIS. When necessary, the brightness and contrast of the resulting images were adjusted with Krita. The schemes were drawn in Inkscape.

## Data Availability

The datasets analyzed during this study are available from the corresponding author on reasonable request. All the necessary data are presented in the paper.
